# Enhanced global carbon cycle sensitivity to tropical temperature linked to internal climate variability

**DOI:** 10.1126/sciadv.adl6155

**Published:** 2024-09-25

**Authors:** Na Li, Sebastian Sippel, Nora Linscheid, Christian Rödenbeck, Alexander J. Winkler, Markus Reichstein, Miguel D. Mahecha, Ana Bastos

**Affiliations:** ^1^ Max Planck Institute for Biogeochemistry, Jena 07745, Germany.; ^2^ Institute of Meteorology, Leipzig University, Leipzig 04103, Germany.; ^3^ Institute for Earth System Science and Remote Sensing, Remote Sensing Centre for Earth System Research (RSC4Earth), Leipzig University, 04103 Leipzig, Germany.

## Abstract

The sensitivity of atmospheric CO_2_ growth rate to tropical temperature (γ^T^) has almost doubled between 1959 and 2011, a trend that has been linked to increasing drought in the tropics. However, γ^T^ has declined since then. Understanding whether these variations in γ^T^ reflect forced changes or internal climate variability in the carbon cycle is crucial for future climate projections. We show that doubling sensitivity events can arise in simulations by Earth system models with perturbed initial conditions but are likely explained by internal climate variability. We show that the doubling sensitivity event is associated with the occurrence of a few, but very strong, El Niño events, such as 1982/83 and 1997/98. Such extreme events result in concurrent carbon release by tropical and extratropical ecosystems, increasing the variance of the global land carbon sink and its apparent sensitivity to tropical temperature. Our results imply that the doubling sensitivity does not necessarily indicate a change in carbon cycle response to climate change.

## Teaser

Doubling sensitivity of atmospheric CO_2_ to tropical temperature is linked to internal climate variability and El Niño events.

## INTRODUCTION

The global atmospheric CO_2_ growth rate (AGR) is an important indicator on how natural and anthropogenic carbon emissions are taken up by the land and ocean (*1*, *2*). Global AGR has large year-to-year variations (*2*, *3*), which are mainly attributable to land carbon sink variability (*2*, *4*), particularly semiarid tropical regions (*5*). Future trends in the global and tropical land carbon sinks are highly uncertain, leading to high uncertainty in future climate projections (*6*).

The sensitivity of AGR to tropical mean annual temperature (MAT, γ^T^) has been proposed as a means to reduce uncertainty in the future response of the tropical carbon sink to climate change (*7*). Recent studies suggested a twofold increase in the sensitivity of interannual variations of AGR to tropical MAT anomalies over the period 1959–2011 (*8*, *9*), interpreted a strengthened global land sink response to climate. We refer to this event as a “doubling sensitivity event.”

Previous studies proposed that the doubling sensitivity event was associated with impacts from increasing extreme tropical droughts (*8*, *9*) in a warming climate. However, climate trends over a few years to decades may evolve because of external forcing, internal climate variability, or both (*10*, *11*). These forcings may cause low-frequency (few years to decades) climate variations (*10*, *11*), and it is important to distinguish such unforced signals from the forced signal of climate change. For instance, the slower increase of global mean surface temperature in the period 1998–2012 (“warming hiatus”) was mainly caused by internal climate variability and naturally forced variability, with a strong El Niño at the beginning of the period and a couple of La Niña events at the end (*12*–*14*).

Variability in the global carbon cycle is known to be strongly driven by internal climate variability, primarily by the El Niño–Southern Oscillation (ENSO) (*15*) as well as other large-scale climatic variability modes (*16*, *17*). For instance, Indian Ocean dipole (*18*), Pacific Decadal Oscillation, and Atlantic Multidecadal Oscillation also influence the land carbon fluxes (*19*, *20*). These modes arise from internal climate variability and emerge on a continuum of seasonal to multidecadal timescales (*14*, *21*). Interannual to multidecadal changes in the climate system thus result from an intricate combination of internal climate variability and external forcing (*22*, *23*), with external forcing the combination of natural forcings (solar input and volcanic eruptions) and anthropogenic forcing (*11*, *21*, *24*–*27*).

Internal climate variability is considered to be irreducible noise of Earth climate system (*11*, *28*). It can mask or enhance external forced climate variations, particularly on decadal and regional scales (*29*–*32*). Because of the stochastic and chaotic nature of atmosphere-ocean variability, even under the same external forcing and physical processes, the realization of internal climate variability can be different (*31*). Accordingly, the observed response of the carbon cycle might be different under the same external forcing (*33*, *34*).

Furthermore, following a peak around 1985 to 2004, γ^T^ showed a decline in 1997 to 2016 (*9*). If the doubling sensitivity event would reflect a long-term response of the carbon cycle to increasing drought forced by climate change, the decrease of γ^T^ in recent years would likely not have happened, especially because increases in global and regional mean temperature and atmospheric dryness have continued in recent years (*35*). We thus hypothesize that the doubling sensitivity might not necessarily reflect a response to forced climate change but may contain contribution from internal climate variability.

A mechanistic understanding of how γ^T^ changes are a crucial prerequisite to assess whether forced climate change or internal climate variability is likely at play in doubling sensitivity events. However, as γ^T^ is estimated on the basis of two large-scale aggregated signals (global AGR and tropical MAT), it might not necessarily reflect local physiological changes in the response of the terrestrial carbon sink to climate variability. At local scale, water availability is the main direct driver of land carbon sink variations, especially in the tropics (Tro) (*36*). Its influence is, however, obscured when aggregating fluxes spatially, with temperature becoming more dominant at continental to global scales (*36*, *37*). A study showed that apparent changes in γ^T^ can arise even if the land carbon sink anomalies are derived from temperature variations based on constant local sensitivities of net carbon uptake to temperature (*38*). Apparent changes in γ^T^ might have resulted partly from shifts in the location of temperature variations and covariability with water availability (*38*).

Previous studies have focused on tropical ecosystems when assessing the doubling sensitivity event (*8*, *9*). This is based on the assumption that tropical land contributes the most to interannual variability in AGR (*5*, *39*). Accordingly, the tropical land should crucially contribute to the doubling sensitivity event. However, given that AGR is a globally integrated metric, one cannot exclude the influence of variations in carbon fluxes in other regional domains. For example, recent studies found that the ocean is more variable than previously acknowledged (*40*, *41*), with important contributions from the extratropics, particularly the Southern Ocean (*40*). Moreover, changes in the sensitivity of the Northern Hemisphere’s (NH’s) seasonal terrestrial carbon sink to temperature have been reported (*42*).

It therefore remains elusive whether the doubling sensitivity event reflects an intrinsic change in the carbon cycle response to climate, or an apparent trend due to spatial and process aggregation as proposed in (*38*), and whether this change is driven by climate change. In this study, we evaluate whether the doubling sensitivity event: (i) may have been driven by internal climate variability or forced climate changes; (ii) reflects a causal change in the sensitivity of the carbon cycle to tropical temperature; and (iii) is a tropical land signal, or is confounded by variability in other land and ocean regions.

## RESULTS

**Fig. 1.**
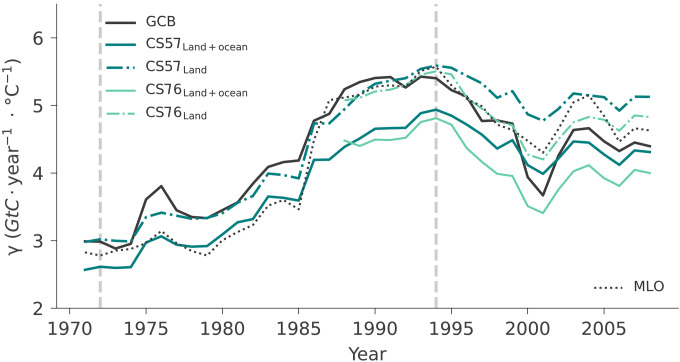
Sensitivity of AGR to tropical MAT over 25-year moving windows (see Materials and Methods) between 1959 and 2020 (γ^T^). The lines show trends in γ^T^ based on atmospheric CO_2_ measurements at Mauna Loa (MLO, black dotted line), as in (*8*), and global mean AGR derived from multiple sites in the Global Carbon Budget 2021 (GCB, black filled line). We compare these with γ^T^ based on global land and ocean CO_2_ fluxes estimated by the Jena CarboScope atmospheric inversion using the stations Barrow, Mauna Loa, and South Pole covering the 1959 to 2020 period (CS57_Land+ocean_, solid teal), and an inversion based on further stations available since at least 1976 (CS76_Land+ocean_, solid light green). In addition, we show γ^T^ calculated only on the basis of the land flux estimates by the two inversions CS57_Land_ and CS76_Land_, (dash-dot lines of respective color). The doubling sensitivity period is derived on the basis of Mauna Loa AGR and ranges from 1972 to 1994 as central years of the 25-year moving window (two vertical gray dashed lines).

We first compare γ^T^ based on AGR from atmospheric measurements at Mauna Loa, as in (*8*), and based on global average AGR from the Global Carbon Budget (GCB) 2021 (Fig. 1, black dotted and filled lines, respectively) (*2*, *43*). Both datasets show a consistent increase in γ^T^ from 1960 to 1984 (2.8 and 3.0 *GtC* · year^−1^ · °C^−1^ for Mauna Loa and GCB, respectively) to 1982 to 2006 (5.6 and 5.4 *GtC* · year^−1^ · °C^−1^). After a peak centered in 1994, γ^T^ drops to 4.6 and 4.4 *GtC* · year^−1^ · °C^−1^ in Mauna Loa and GCB, respectively. This corresponds to a decrease of 18 to 19%.

While both datasets show a consistent increase in γ^T^, they show important differences in the beginning and end of the study period. These differences can be due to the fact that AGR calculated from CO_2_ mole fractions at Mauna Loa is influenced by land and ocean CO_2_ fluxes but also by variations in atmospheric transport (*44*). Conversely, $\gamma_{\mathrm{GCB}}^{T}$ might be influenced by variability in the number of sites considered. To control for these effects, we estimate γ^T^ on the basis of the sum of global land and ocean carbon fluxes (i.e., comparable to the global AGR signal) from two Jena CarboScope inversions (see Materials and Methods) (*45*) covering different periods with a fixed number of sites: CS57, covering the period of 1959 to 2020 based on three sites, and CS76, covering 1976 to 2020 and based on nine sites (CS57_Land+ocean_ and CS76_Land+ocean_; Fig. 1, solid teal and light green). Both inversions reproduce the doubling sensitivity event and show very similar sensitivity variations to those of $\gamma_{\mathrm{GCB}}^{T}$ , but they estimate a weaker increase than $\gamma_{\mathrm{MLO}}^{T}$ . Transport effects seem to partly explain these differences, as we can well reproduce $\gamma_{\mathrm{MLO}}^{T}$ when transporting the fluxes from CS57_Land+ocean_ forward with its atmospheric model (see fig. S2).

Since the two inversions well reproduce γ^T^, we then isolate the contribution of the land to γ^T^ based on the land CO_2_ fluxes from the two inversions (teal and light green, dash dot lines in Fig. 1). The sensitivity due to land fluxes alone is larger than that because of the sum of land and ocean fluxes in both CS57 and CS76. This is consistent with the fact that interannual variations in land and ocean fluxes are partly anticorrelated (*46*) but also indicates that the land sink contributes the most to the doubling sensitivity event.

To avoid introducing possibly spurious variations due to the averaging of records from multiple stations as in GCB, and to keep consistency with (*8*), we define the doubling sensitivity event based on $\gamma_{\mathrm{MLO}}^{T}$ . The event occurs from 1972 to 1994 (Fig. 1), corresponding to the centers of the corresponding 25-year intervals covering 1960 to 1984 and 1982 to 2006, respectively (see Materials and Methods). Given that the land carbon sink contributes the most to the doubling sensitivity, in the remainder of this study, we focus mostly on γ^T^ based on the land sink, using the estimates from the CS57 atmospheric inversion as reference, given its full coverage of the relevant study period.

### Doubling sensitivity events arise in EMS large-ensemble simulations

Internal climate variability can be separated from forced signals by using single or multiple Earth system models (ESMs) initial-condition large ensembles (*22*, *31*), i.e., a large number of simulations with different initial conditions (atmospheric conditions and/or ocean states). Under the same physical process representation and historical forcing, the ensemble mean of all realizations is considered as the forced response (*11*), i.e., changes driven by external forcing to the climate system. The residuals of the individual runs from the ensemble mean are considered as realizations of internal climate variability, i.e., stochastic variations in the weather and climate system (*11*, *22*). Interactions between forced and internal components may exist but are usually assumed small to first order (*11*, *28*). The external forced signal emerges as a change in the ensemble mean, and the residual after subtracting the ensemble mean is considered to reflect internal climate variability (*31*). Large ensembles thus offer valuable insights for interpreting trends and variability in the observed climate record.

**Fig. 2.**
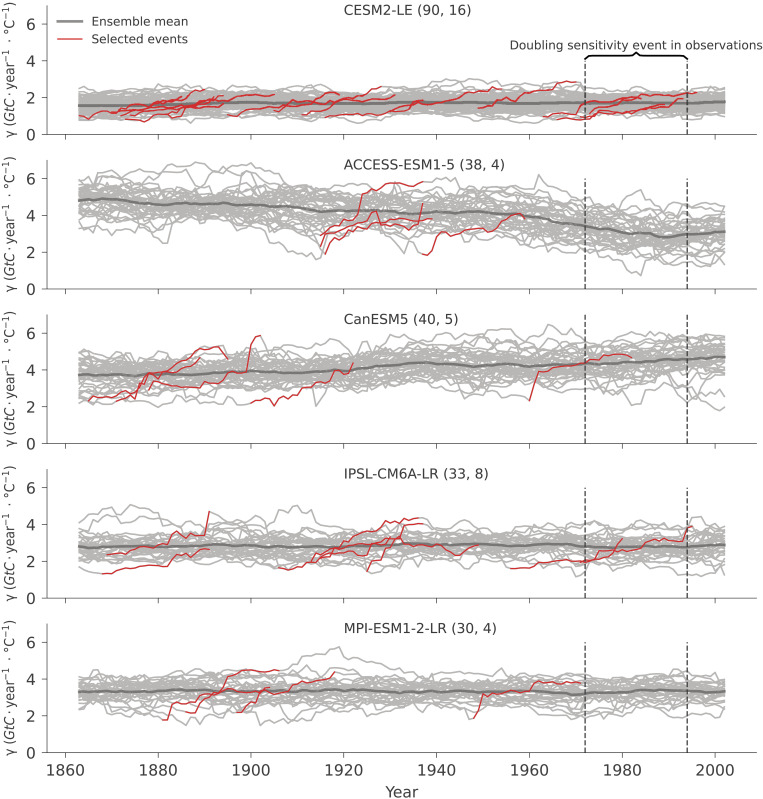
The sensitivity of net land carbon sink NBP to tropical MAT based on individual realizations from five ESM large ensembles (individual panels) in the period 1851 to 2014 (gray lines), over 25-year moving windows. The historic doubling sensitivity event period 1972 to 1994 is plotted between the two vertical dashed lines. The number of simulations included and the number of selected events of each ESM are listed after the model name in the legend. As in Fig. 1, the year in the *x* axis is the center of each 25-year interval. The ensemble mean sensitivity of each model is shown in a thick gray line. The doubling sensitivity events in individual members are emphasized by red lines. For sign consistency with AGR, NBP is multiplied by −1 in the calculation of γ^T^.

To test whether the doubling sensitivity events can be reproduced by ESMs, we analyze the sensitivity of global net biome production (NBP) to tropical MAT in historical simulations by ESM initial condition ensembles. We select five models that include at least 30 realizations (see Materials and Methods) to ensure that the distribution of internal climate variability is well sampled (*47*). In Fig. 2, we show the evolution of the sensitivity of NBP to tropical MAT simulated by each ESM. The models show differences in the absolute magnitude of γ^T^ (see in-depth discussion in Materials and Methods). All ESMs show strong variability across individual realizations in the period 1851 to 2014, suggesting a strong internal variability component in γ^T^ trends. The ensemble mean, corresponding to the forced component, show no notable trends over the full 1851 to 2014 period for all models except Australian Community Climate and Earth System Simulator (ACCESS), which shows a declining trend.

While this could imply that ESMs are not able to simulate doubling sensitivity events, we find that all ESMs include realizations where the doubling sensitivity occurs, although these events are relatively rare (Fig. 2, red lines). The numbers of selected similar events is different among ESMs, but the frequency of doubling sensitivity events is comparable (Fig. 2; number of all realizations and selected similar events are listed after the model name). No consistent temporal patterns for the occurrence of the doubling sensitivity events can be found, which is an indication that doubling sensitivity events are likely to be generated by internal climate variability.

### Doubling sensitivity events are linked with strong El Niño events

**Fig. 3.**
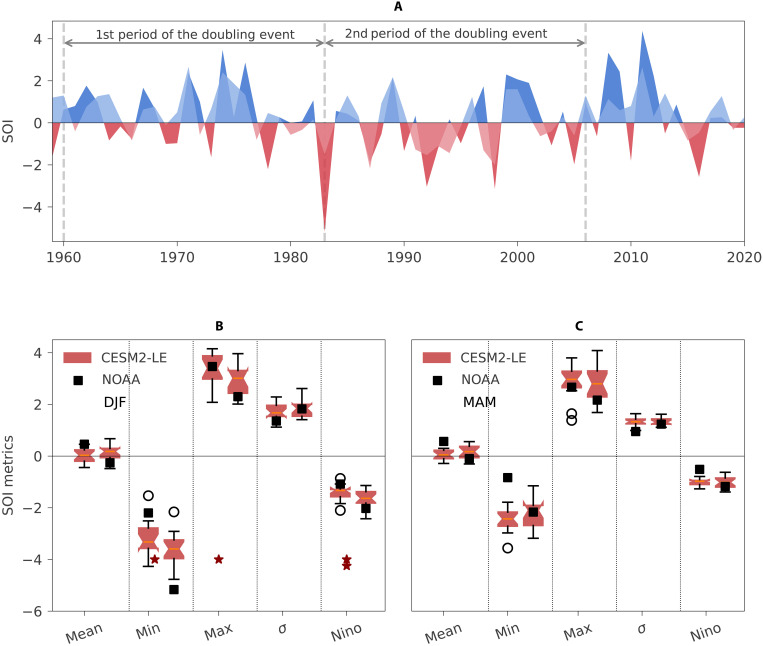
Comparison of seasonal SOI time series trend in observation (NOAA) and 16 selected events in CESM2-LE. (**A**) Seasonal SOI time series from NOAA observation in a 25-year moving window. DJF is dark blue above zero and dark red below zero, MAM is in light blue above zero and light red below zero. (**B** and **C**) compares the trend of SOI time series in DJF and MAM separately. During the doubling sensitivity period, we calculate the mean, minimum, maximum, standard deviation, and Nino phase [mean(SOI) if SOI < 0] of SOI time series (corresponding to mean, min, max, σ, and Nino in the *x* axis). We calculate the above metrics for the first and second half period of each doubling sensitivity event, corresponding to the two boxplots (distribution across 16 events in CESM2-LE) and two black squares (SOI NOAA) shown for each metric. For the observations, the first (1960 to 1982) and second (1983 to 2006) halves correspond to the two periods in the beginning and the end of the doubling sensitivity event (1960 to 2006; Fig. 1). For the CESM2-LE time series, we perform the same split but considering the period covered by each individual event in each model realization. We then use a paired Wilcoxon signed-rank test to evaluate whether the medians of SOI metrics in the second half of each event differ from the corresponding first half (red stars, 1 star for *P* < 0.1 and 2 stars for *P* < 0.05).

As the doubling sensitivity events are likely to be explained by internal climate variability, here, we investigate potential underlying driving mechanisms. Given the predominance of ENSO in influencing the land carbon sink variability (*15*, *17*, *48*), we analyze the links between the doubling sensitivity events and ENSO in more detail. The warm and cold phases of ENSO (El Niño and La Niña) are known to have asymmetric impacts on regional climate anomalies (*49*, *50*): El Niño events are predominantly associated with fast release fluxes (drought impacts and fires), while La Niña events are associated with regrowth fluxes (*51*, *52*). In the historical record, El Niño events have been associated with stronger land source anomalies than the sink anomalies during La Niña events (*53*). Given that ENSO displays multidecadal variability (*14*), it is expected that periods dominated by warm phases should correspond to different carbon cycle variability patterns than periods dominated by cold phases. In particular, the period in the 1950s to mid-1970s was characterized by more and stronger La Niña events, while the mid-1970s until ~2000 registered several extremely strong El Niño events, such as 1982/83 and 1997/98 (Fig. 3A). We further note that the moving window approach to derive γ^T^ is especially sensitive to outliers in the time series. We focus on the two seasons that typically induce stronger carbon cycle impacts, December to February (DJF) and March to May (MAM) (*17*). The smoothed average Southern Oscillation Index (SOI) time series for both seasons show multidecadal difference (fig. S5). With an increase from predominantly positive SOI phases (La Niña conditions), especially for MAM in the 1970s, to negative SOI phases (El Niño) in the late 1980s to 1990s, followed by a decline afterward (fig. S5). These trends fit well with those in γ^T^ during and after the doubling sensitivity, with the smoothed MAM SOI time series correlating strongly (−0.79 to −0.92) with the various γ^T^ time series (table S2).

We then evaluate whether the trends in the smoothed SOI time series during the doubling sensitivity event are influenced by: (i) changes in the mean phase, (ii) the occurrence of extreme El Niño (minimum SOI) or La-Niña (maximum SOI) events, (iii) changes in the variance of SOI (standard deviation), or (iv) stronger and longer El Niño phases (integral of negative SOI), shown in Fig. 3 (B and C). In the observations, and for both DJF and MAM seasons, we find very small differences in the mean and variance of SOI between the first and the second half of the doubling sensitivity periods. We find a slightly stronger El Niño signal (minimum SOI; Fig. 3, B and C, black squares) in the second half of the event but the largest differences related to the individual ENSO events: The strongest El Niño in the second half of the doubling sensitivity event is considerably more intense (SOI = −5.2 in DJF and −2.2 in MAM) than in the first half of the event (SOI = −2.2 in DJF and −0.8 in MAM), and the strongest La-Niña is weaker in the second half of the event than in the first half (Fig. 3, B and C, black squares).

If a few extreme events clustering in a period shorter than the length of the moving window applied can influence γ^T^, it is possible that many other event types could contribute to such doubling sensitivity events, e.g., volcanic eruptions. Therefore, we evaluate whether ENSO also plays an important role in doubling sensitivity events in Community Earth System Model version 2–large ensembles (CESM2-LE), which has the largest number of events from the five ESMs considered. In CESM2-LE, the distributions of the SOI metrics for the 16 doubling sensitivity events are generally consistent with the observations, especially in DJF, when stronger extreme El Niño and weaker extreme La Niña and significantly more prevalent El Niño conditions in the second half of the doubling sensitivity events than in the first. For MAM, however, no significant differences in SOI metrics simulated by CESM2-LE between the two halves of the doubling sensitivity events are found. CESM2-LE has been shown to simulate well the seasonal evolution of ENSO events (*54*), but we cannot exclude uncertainties on the lagged temporal responses of the carbon cycle to ENSO events (*20*) or confounding effects by other modes of variability in the observations. Nevertheless, the results indicate that the CESM2-LE allows us to capture internally generated doubling sensitivity events for similar reasons as in the observations. This is the evidence that the event is driven by few extreme events associated with internal climate variability.

### Increased AGR standard deviation dominates the twofold-enhanced sensitivity

We show that the doubling sensitivity event is explained by trends in γ^T^ that appear to be associated with few extreme events induced by internal climate variability. It remains unclear whether variations in γ^T^ really reflect systematic and mechanistic changes in the sensitivity of the land carbon sink to climate due to such extreme events. The sensitivity (linear regression slope) depends on the ratio of the sample standard deviations, multiplied by their Pearson correlation coefficient as

$$\gamma^{T}=\frac{\text{dAGR}}{\text{dMAT}}=cor\;\left(\mathrm{AGR},MAT\right)\;\frac{std\left(\mathrm{AGR}\right)}{std\left(\mathrm{MAT}\right)}$$ (1)

**Fig. 4.**
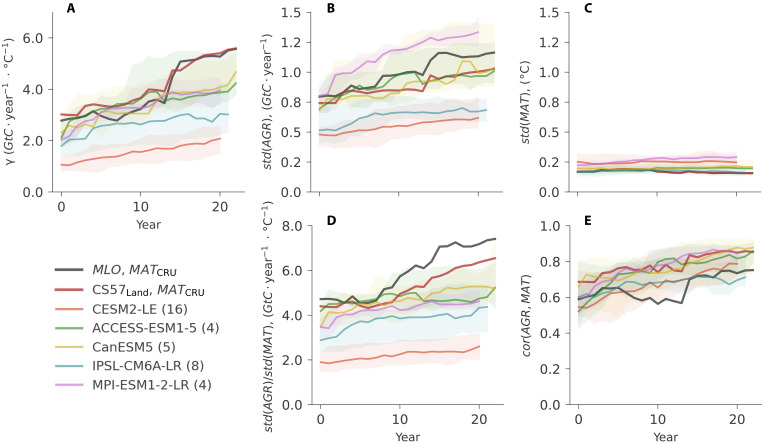
Statistical analysis of doubling sensitivity of Mauna Loa AGR (MLO, black lines), Jena CarboScope CS57 land sink (CS57_Land_, red lines), and selected similar events from five ESM large ensembles (colored lines and corresponding shades), over 25-year moving windows. (**A**) The doubling sensitivity and all the similar events from the five ESMs are plotted. The statistical analysis includes the standard deviation of AGR and tropical MAT, represented by *std*(*AGR*) and *std*(*MAT*), respectively (**B** and **C**); relative variation *std*(*AGR*)/*std*(*MAT*) (**D**); and correlation *cor*(*AGR*, *MAT*) (**E**). The selected events from the ESM large ensembles are represented by median and 5 to 95% quantile (colored lines and corresponding shades). The number of selected events is listed behind each model name in the legend. Note that in ESM large ensembles, we use NBP instead of AGR, and NBP has been multiplied by −1 for sign consistency to AGR. The year in the *x* axis is the center of each 25-year moving window. The selected events in ESM large ensembles arise at random times and are not synchronized with the doubling sensitivity in Mauna Loa (Fig. 2, red lines).

Therefore, a doubling of γ^T^ could be due to an increase in the correlation between AGR and MAT, an increase in the variance of AGR, a decrease in the variance of MAT, or a combination of these each factor to the increase in γ^T^ during the doubling sensitivity event for observations ( $\gamma_{\mathrm{MLO}}^{T}$ , $\gamma_{\mathrm{CS}57}^{T}$ ), and ESMs (Fig. 4).

We thus calculate the relative change of each term during the doubling sensitivity event (Materials and Methods and Eq. 5), as an approximation of their individual contributions to the change in γ^T^. We find that the standard deviation of AGR (Fig. 4B) shows the largest relative changes during the doubling sensitivity for both $\gamma_{\mathrm{MLO}}^{T}$ and $\gamma_{\mathrm{CS}57}^{T}$ , with the standard deviation of MAT (Fig. 4C) remaining roughly stable over the period and the correlation between the two variables increases slightly (Fig. 4E). *std*(AGR_MLO_) changes from 0.80 to 1.16 *GtC* · year^−1^ in 1972 and 1994 (0.74 to 1.03 for CS57), in the 25-year moving window. While the correlation between AGR_MLO_ and MAT increases from 0.59 to 0.75 (0.69 and 0.85 in CS57). The change in the variation ratio between AGR and MAT dominates the doubling sensitivity event (Fig. 4D), which correspond approximately to 57 and 49% of $\gamma_{\mathrm{MLO}}^{T}$ and $\gamma_{\mathrm{CS}57}^{T}$ change. By contrast, the correlation term contributes only by ~28 and 24% to $\gamma_{\mathrm{MLO}}^{T}$ and $\gamma_{\mathrm{CS}57}^{T}$ change, respectively (table S3).

We then analyze relative changes in each term contributing to γ^T^ estimated by ESMs (Fig. 4, colored lines and shades). Depending on whether the doubling sensitivity events are driven by a single phenomenon, or potentially induced by different phenomena affecting each of these terms, the decomposition of γ^T^ in observations does not necessarily have to be the same as in each realization of ESMs but expected to be comparable if the models can represent the underlying mechanisms. As shown in Fig. 2, the absolute magnitude of γ^T^ differs across models, particularly for CESM2-LE (Fig. 4A), which is mostly due to differences in the magnitude of variability in NBP. The standard deviation of MAT and correlation of NBP with MAT are consistent with both AGR_MLO_ and CS57. The individual terms of the statistical relationship show variability across individual events (shaded areas in Fig. 4, summarized fig. S6), but, in general, the ESMs agree on the relative changes in the different terms in Eq. 1, with increases in *std*(*NBP*) across all events and increases in *cor*(*NBP*, *MAT*) for most CM6A-LR and Canadian Centre for Climate Modeling and Analysis (CanESM5) and all events in the other ESMs (fig. S6). The relative changes in each term in MLO and CS57 are in most cases within the spread across individual events in ESMs, excepting the increase in correlation term for ACCESS-ESM1-5 and Max Planck Institute for Meteorology (MPI)–ESM1-2-LR, which is stronger than in observations. While the relative changes in the different terms generally follow the pattern that we find in observations, each individual doubling sensitivity event is—to some extent—unique (tables S4 to S8). Nevertheless, we find a few individual events in ESMs that show very similar relative changes in each component to those in the observations, for example, events 2, 3, 9, and 11 in CESM2-LE, event 3 by ACCESS-ESM1-5, events 2 and 5 in CanESM5, events 2, 5, and 7 in IPSL-CM6A-LR, and event 2 of MPI-ESM1-2-LR. These results further highlight the semi-stochastic nature of doubling sensitivity events.

Both an increase in the standard deviation of AGR or of the global land sink correlation with temperature would be consistent with the well-established and important role of ENSO in dominating variability of the land sink. The increase in the correlation could hint at changes in the ecophysiological mechanisms underlying the relationship between the land carbon sink and MAT, while the increase in standard deviation of AGR and NBP beyond that of temperature could indicate a nonlinear increase in carbon release during stronger El Niño events or arise from changes in regional contributions to the variance in AGR. A previous study in (*55*) showed that an atmospheric inversion with fixed and season-specific local linear sensitivities to temperature could still predict extreme anomalies during the strongest El Niño events based only on information from weak El Niño and normal La Niña variability, and capture a large fraction of the increase in sensitivity of the global land sink to MAT. This suggests that the doubling sensitivity event more likely reflects an apparent sensitivity change rather than large-scale ecophysiological changes in the relationship between the land-sink and ENSO.

**Fig. 5.**
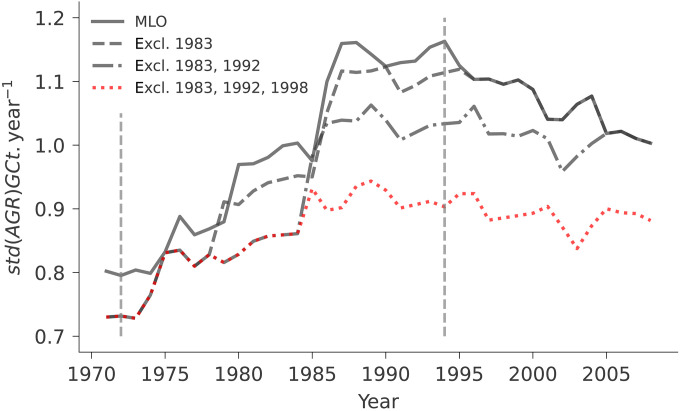
Standard deviation variation of AGR from Mauna Loa in 25-year moving windows when removing different strong El Niño years. Note that “excl.1983” means that 1983 was removed from the time series, and “excl. 1983, 1992” means that the years 1983 and 1992 were removed from the time series at the same time.

An apparent sensitivity change could thus be due to multiple factors, such as changes in the relative contributions of different regions or seasons to *std*(*AGR*). However, the trend in *std*(*AGR*) might also simply result from a lever effect of individual extremes on time series that become evident when performing moving window calculations. We find that the trend in $\gamma_{\mathrm{MLO}}^{T}$ is extremely sensitive to three individual years: 1983, 1992, and 1998. Each of these individual years induces a sharp increase in the *std*(*AGR*) in Mauna Loa subsequently (Fig. 5) when it is included for the first time in the 25-year moving window and its influence persists over the next 25 years. All 3 years correspond to interannual extremes in the carbon cycle: the three most intense El Niño events on record, 1982/3, 1991/2, and 1997/8 (Fig. 3A). While the impact of the 1991/2 El Niño was masked by the influence of the Mt. Pinatubo volcanic eruption in 1991, both 1982/3 and 1997/8 have been associated with extreme carbon emissions due to droughts and fires (*46*, *55*, *56*). The individual effects of each extreme year are then further accumulated over time through the application of the moving window, resulting in an increasing *std*(*AGR*) trend in Mauna Loa: During the doubling sensitivity period, the removal of these 3 years from the 62-year time series results in an almost halving of the *std*(*AGR*) relative change, from 46 to 26% (Fig. 5).

### NH also contributes to the doubling sensitivity

Given that the doubling sensitivity appears to be explained by few events that typically have global scope, it is likely that other regions beyond the tropical land ecosystems play a role in γ^T^ changes. First, given that ENSO has opposing impacts on the land and ocean sinks (*46*), we decompose the change in AGR variance into land and ocean components by CS57 (fig. S7 and see Materials and Methods, Eq. 7). This decomposition shows that the land is the main contributor to the increase in AGR variance, contributing by 103% to its change in the doubling sensitivity period (note that values over 100% mean that another term contributes negatively). The variance of the ocean sink also increases slightly (13%) during this period, with the changes in the individual sinks being counteracted by a strengthening of the negative covariance between land and ocean (*46*).

**Fig. 6.**
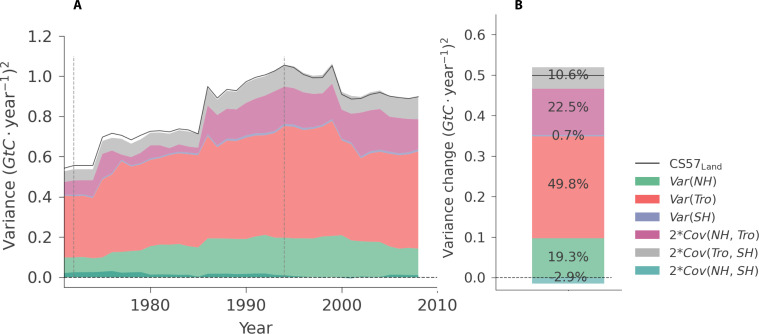
Variance decomposition of global land sink (land to atmosphere) from Jena CarboScope (CS57_Land_) over 25-year moving windows. (**A**) Global land sink variance decomposed to different spatial domains: Northern Hemisphere (NH), Tropics (Tro), and Southern Hemisphere (SH). (**B**) The fraction contributed by each component in (A) to the global land sink variance change. On the basis of the variance change of each component in the doubling sensitivity period (variance at the end of the event minus variance at the start of the event, see Materials and Methods). The doubling sensitivity period is 1972 to 1994 (two vertical dashed lines). The variance is represented as “Var” and covariance as “Cov.”

Then, we evaluate regional contributions to the change in variability of the land sink by decomposing the variance of NBP from CS57_Land_ into NH, Tro, and Southern Hemisphere (SH) (Fig. 6A; see Materials and Methods, Eq. 8). To compare the contribution of each domain to the doubling sensitivity event, we calculate the change in each term of the variance for each individual domain between the start and the end of the doubling event (Fig. 6B). We also decompose NBP from CS76_Land_ since it includes more atmospheric measurement stations (9) during the 1976 to 2020 period (fig. S8).

We find that the Tro and NH land ecosystems are the main contributors to the variance change of the global land sink during the doubling sensitivity period (Fig. 6). The Tro alone contribute 49.8% of the total global land variance change, the NH alone contributes 19.3%, while an increase in the covariance between the NH and tropical sinks contributes 22.5% (Fig. 6B). The SH and its covariance with the tropical sink together contribute marginally to the change, 11.3% (Fig. 6B), likely due to the smaller extent of land in the SH. Hence, other than tropical land sink alone, the NH also plays an important role to the increase in global AGR variance during the doubling sensitivity event.

**Fig. 7.**
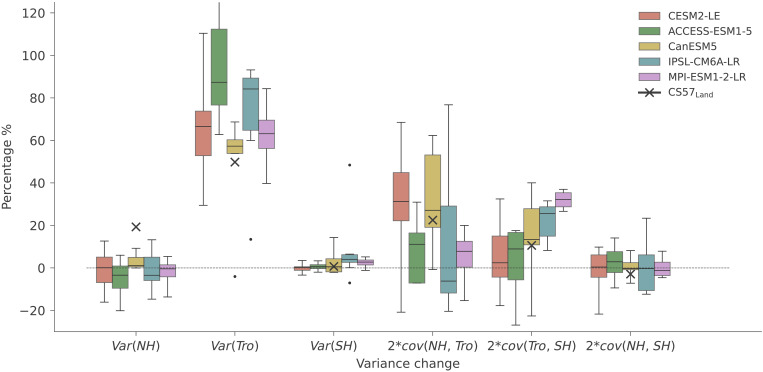
Contribution of each spatial domain (NH, Tro, and SH) to the variance change of global land sink during the doubling sensitivity period. We compare the contribution of each domain in Jena CarboScope CS57 (CS57_Land_ black cross) and ESMs, calculated from the same values as in Fig. 4B with selected doubling events in five ESM large ensembles (colored boxes).

To test whether these results agree with ESMs, we then decompose the NBP variance of the doubling sensitivity events in ESM large ensembles (selected events in Fig. 4). We compare the contribution of each decomposed variance of NBP from CS57_Land_ to the events simulated by the five ESM large ensembles (Fig. 7). The ESMs attribute a stronger contribution of the variance in the Tro than CS57_Land_ and only residual contributions of the NH variance, while the covariance terms of NH and SH with the tropical sink are roughly consistent with CS57_Land_ (Fig. 7). This mismatch with CS57_Land_ is likely explained by the fact that land surface models typically assign larger variability to the tropical regions than atmospheric inversions and data-driven products (*39*), specifically during El Niño events (*16*).

## DISCUSSION

This study revisits the suggested twofold-enhanced sensitivity of global AGR to tropical MAT (*8*, *9*). We show that the doubling sensitivity does not necessarily reflect a causal change in the carbon cycle response to temperature but appears to be an apparent change explained by the occurrence of few extreme El Niño events clustered in the 1980s and 1990s. When estimating sensitivity changes based on a multidecadal moving window, strong and long-lasting La Niña events at the beginning of the event and the emergence of few but strong El Niño events result in a lever effect that pushes the sensitivity to its peak, declining subsequently. We show that this increase is associated with an increase in the variance of the global land sink: Extreme El Niño events result in fast releases of carbon to the atmosphere due to drought and heat anomalies in tropical and some extratropical ecosystems (*16*, *46*, *51*, *57*). These differences in land sink variance between the two periods of the doubling sensitivity event can be explained by the “slow-in, fast-out” nature of land carbon cycle, as well as with regional differences in the global impacts of El Niño versus La Niña (*49*, *50*, *58*), and by changes in regional contributions to AGR variability.

Given their extreme magnitude, these few El Niño events are expected to result in global impacts. This is reflected in an increased covariance between the tropical and extratropical sinks in both observation-based datasets and ESMs, occurring especially when each El Niño event is entering in the 25-year moving window (Fig. 7). Nonetheless, we note that constraining regional contributions to global land sink variance is still a major challenge (*2*) so that uncovering the regional driving mechanisms is still prone to large uncertainties (*36*, *59*). However, in both observations and ESMs, we found the doubling events linked to changes in large-scale ocean-atmospheric circulation (*15*–*17*). Variations in extreme events are likely associated with slowly evolving patterns of internal climate variability. ESMs can reproduce multiple doubling sensitivity events roughly consistent with observations in the period 1851 to 2014, without a forced signal component. Moreover, each individual event in ESMs is unique in terms of changes in the variance in the global land sink, tropical MAT, their correlation, and in the regional contributions to those changes. This emphasizes the importance of accounting for uncertainty driven by internal climate variability when assessing recent trends in climatic (*12*, *13*) and carbon cycle variables (*17*, *34*).

Our results indicate that studies on the sensitivity of the global AGR to climate variations, and especially its changes over time, require reexamination. First, we show that the trend in γ^T^ is partly explained by statistical artifacts due to the presence of clustered outliers in the time series. Second, we show that the NH also plays an important role in changes in the global AGR variability, not just the tropical land (Figs. 6 and 7), i.e., the doubling sensitivity event reflects a change in apparent sensitivity, rather than in intrinsic ecophysiological sensitivity. Hence, we conclude that the change in “sensitivity” as given by the regression slope of two highly integrated metrics such as global AGR and annual mean tropical temperature does not reflect the local ecosystem response to the direct drivers (*36*, *38*, *39*, *60*) nor does it imply a mechanistic change in global carbon cycle sensitivity to climate, as suggested, e.g., in (*9*).

Trends over multiple decades in aggregated carbon cycle metrics, such as the AGR sensitivity to temperature, must therefore be interpreted carefully. This is especially relevant when constraining ESM projections of future carbon cycle based on such metrics, e.g., (*7*), as they contain components of internal climate variability and externally forced responses. In a broader sense, these results imply that the role of internal climate variability on trends in the carbon cycle at decadal timescales requires further scrutiny. A mechanistic understanding of the links between the carbon cycle and internal and forced components of climate variability is crucial for robust attribution of impacts and trends (*22*, *61*). Particularly relevant for the interpretation of future projections is that those components of recent historical trends driven by internal climate variability will change or even reverse. The forced components of recent trends in the carbon cycle are likely to continue to increase with future climate change.

## MATERIALS AND METHODS

### Experimental design

We compare the observations and atmospheric inversions in the period 1959 to 2020 and five ESM large ensembles (*62*–*64*) in the period 1851 to 2014. Here, we investigate (i) whether the ESM large ensembles show a similar doubling sensitivity as in observations and whether the trend can be generated by internal climate variability alone, (ii) how the ENSO signal SOI index is linked to the doubling sensitivity, (iii) the mathematical drivers of the doubling sensitivity, and (iv) the dominant spatial contributors to the global AGR variance change: We decompose the global land carbon variations to different spatial domains, NH, Tro, and SH. We then compare the contribution of each spatial domain to global land carbon variation changes over the doubling sensitivity event.

### Datasets

#### Atmospheric CO_2_ growth rate

We use the Mauna Loa AGR datasets for 1959 to 2020 from the Scripps Institution of Oceanography, and National Oceanic and Atmospheric Administration (NOAA) Global Monitoring Laboratory, Mauna Loa Observatory, Hawaii (*3*, *65*). The data have been converted to a CO_2_ flux assuming instantaneous atmospheric mixing. The AGR in unit of *ppm* · year^−1^ is converted to unit of *GtC* · year^−1^ by multiplying by a factor of 2.124 (https://gml.noaa.gov/webdata/ccgg/trends/co2/co2_gr_mlo.txt; last accessed on 19 November 2022).

We also use global AGR from the GCB 2021 for 1959 to 2020 (*2*). The global AGR is provided by the US NOAA Earth System Research Laboratory (*43*) and then later updated (*66*) and recently revised (*67*). For the period 1959 to 1979, the CO_2_ concentration is based on the averaged measurements from the Mauna Loa and South Pole stations, as observed at Scripps Institution of Oceanography (*3*). For the period 1980 to 2020, the CO_2_ concentration is based on the averaged measurements from multiple stations (https://icos-cp.eu/science-and-impact/global-carbon-budget/2021; last accessed on 15 March 2022) (*66*, *68*).

#### Atmospheric inversions

We further use data-based gridded CO_2_ flux estimates from the Jena CarboScope atmospheric inversion, run s57Noc_STD1TneeI_v2022, short for CS57 (https://bgc-jena.mpg.de/CarboScope/?ID=s57Noc_STD1TneeI_v2022; last access on 20 July 2023) (*38*, *45*). This inversion has been constrained by atmospheric CO_2_ data from three stations: Point Barrow supplemented by measurements on ice floes in the earliest years, Mauna Loa, and South Pole (*3*, *65*, *69*). Measurements at these stations are available over essentially all the 1957 to 2021 period, although several gaps exist in the first decades. The inversion setup essentially follows the standard CarboScope set-up version v2022 [(*38*), updated], except that the a priori uncertainties of the interannual degrees of freedom have been weighted spatially. The weighting is according to the temporal standard deviation of the interannual variations of the CarboScope NEE (Net Ecosystem Exchange)-T inversion (run sEXTocNEET_v2022), having interannual variations of the CO_2_ flux proportional to those of local air temperature, scaled by sensitivities based on 196 atmospheric CO_2_ measurement stations (*38*). In addition, the a priori uncertainty of the global flux has slightly been increased (by a factor 1.825). This weighting allows more freedom particularly for tropical CO_2_ flux variations to compensate for the weak overall data constraint from only three stations. Fossil fuel CO_2_ emissions and the ocean CO_2_ flux have been prescribed from GridFEDv2022.2 (updated) (*70*) and an interpolation of surface-ocean pCO_2_ data (CarboScope run oc_v2022, updated) (*71*), respectively. In comparison, we also included another Jena CarboScope atmospheric inversion, run s76oc_v2022, short for CS76 (*38*, *45*) (https://bgc-jena.mpg.de/CarboScope/?ID=s76oc_v2022; last access on 14 August 2023). This standard inversion has been constrained by atmospheric CO_2_ data from nine stations. Measurements are temporally consistent over all the 1976 to 2021 period. We also include a forward run predicted Mauna Loa AGR to evaluate whether CS57 can reproduce the doubling sensitivity by transporting the corresponding surface fluxes forward with an atmospheric model. Note that in atmospheric inversions, we use land-to-atmosphere CO_2_ flux to represent (negative) land sink; the sign of the (negative) land sink is the same as the sign of AGR.

In comparison, we also included another two Jena CarboScope atmospheric inversions: run s85oc_v2022 [here called CS85 (*45*); https://bgc-jena.mpg.de/CarboScope/s/s85oc_v2022.html; last access on 24 July 2023] and run s93oc_v2022 [here called CS93 (*45*) https://bgc-jena.mpg.de/CarboScope/s/s93oc_v2022.html; last access on 24 July 2023]. The two inversions have been constrained by atmospheric CO_2_ data from 21 and 35 stations, respectively.

#### Climate data

Temperature for 1959 to 2020 is from gridded Climatic Research Unit (CRU) data version 4.05 (*72*). The monthly mean data with a resolution of 0.5° × 0.5°, provided by CRU at the University of East Anglia (https://crudata.uea.ac.uk/cru/data/hrg/cru_ts_4.05/; last accessed on 08 December 2021). We use monthly SOI from NOAA (*73*) for the period 1959 to 2020 (https://cpc.ncep.noaa.gov/data/indices/soi, we use the “ANOMALY” data; last accessed on 01 August 2023).

#### Earth system models

We select five ESM large ensembles, one from CESM2-LE (*64*, *74*) and four from CMIP6 (*62*, *63*). For each model, we select variables of NBP (NBP; monthly mean for CESM2-LE and annual mean for CMIP6), monthly mean air temperature at 2 m above surface, all covering 1851 to 2014. All model runs are under historical forcing with coupled land-atmosphere interaction and including biogeochemical cycles. The CESM2-LE (*64*, *74*) is from the CESM2 Large Ensemble Community Project and with supercomputing resources from IBS Center for Climate Physics in South Korea. CESM2-LE consists of 90 members at resolution of 0.9375° × 1.25°, covering the period 1851 to 2014. Under CMIP6 historical runs (*62*), the large ensembles are generated with different oceanic and atmospheric initial settings. Note that the last 40 realizations run with slightly different smoothed biomass burning fluxes, and this leads to stronger variability in biomass burning emissions from 1990 to 2020 (*64*). NBP is downloaded from https://earthsystemgrid.org/dataset/ucar.cgd.cesm2le.lnd.proc.monthly_ave.NBP.html; last accessed on 20 November 2022. Temperature is downloaded from https://earthsystemgrid.org/dataset/ucar.cgd.cesm2le.lnd.proc.monthly_ave.TSA.html; last accessed on 20 November 2022.

Four ESMs from CMIP6 (*62*) include: ACCESS-ESM1-5; (*75*), with 38 realizations; the CanESM5 (*76*) with 40 realizations; the model developed by IPSL [IPSL-CM6A-LR (*77*)], with 33 realizations; and the Max Planck Institute for Meteorology–developed ESM [MPI-ESM1-2-LR; (*78*)] with 30 realizations. The models prescribe the anthropogenic sources of CO_2_ and predict the CO_2_ concentrations (*62*). The simulations are run under historical forcing based on observations, imposed with evolving external forcings such as solar radiation, volcanic activities, and human-caused changes in atmospheric composition (*62*). The datasets are originally from https://esgf-node.llnl.gov/projects/cmip6/ and then collectively aggregated to resolution 2.5° × 2.5° and resampled to annual mean (*63*), last accessed on 25 February 2023.

The seasonal SOI index from CESM2-LE (*79*) was downloaded from https://www.cesm.ucar.edu/projects/cvdp-le/data-repository (scroll to “CESM Comparisons,” and click the “Data” link corresponding to “CESM2 Large Ensemble 1850-2100”) last accessed on 04 February 2022. The SOI index in CESM2-LE is the difference between the Indian Ocean/Western Pacific (E70° to E170°) and the Central/Eastern Pacific (W160° to W60°) and then averaged over the latitude band S30° to 0° [see (*80*)]. The sign of SOI from CESM2-LE is the opposite with SOI from NOAA. For sign consistency, we here invert SOI from CESM2-LE by multiplying by −1.

### Data pretreatment

AGR from Mauna Loa and GCB2021 both have long-term trends removed through locally weighted smoothing [LOWESS; (*81*)], with window size 0.25. We use the LOWESS python package by A. Gramfort (https://gist.github.com/agramfort/850437; last access on 03 April 2023).

The atmospheric inversion Jena CarboScope versions are pretreated as: For each pixel, the time series is resampled to annual sum and then aggregated to the sum of various spatial domains (global, NH, Tro, and SH). Each domain time series has the long-term trend removed by LOWESS (*81*) with window size 0.25.

Tropical MAT anomalies from CRU are pretreated as follows: First, the land spatial domain of N23.5° to S23.5° was selected, and the monthly data were resampled to the annual mean. Then, the area-weighted tropical mean was taken, and the long-term trend was removed by using LOWES (*81*) as done for AGR.

Global annual sum NBP from the five ESM large ensembles are pretreated as follows: For each pixel, the monthly mean time series is resampled to the annual sum for CESM2-LE; the other four CMIP6 models have annual mean aggregated to annual sum, and then the area-weighted sum of various spatial domains is taken: global, NH (N23.5° to N90°), Tro (N23.5° to S23.5°), and SH (S23.5° to S90°). Note that we use a common land-ocean mask for the four CMIP6 models at 2.5° × 2.5° resolution, derived from ESACCI landcover (*82*). For tropical MAT: First, the land domain N23.5° to S23.5° is selected, and then for each pixel, the time series is resampled to the annual mean. This is only for CESM2-LE; the other four CMIP6 models have been aggregated to annual mean in (*63*). Then, the area-weighted mean of the selected tropical domain is taken. For each realization time series, we then detrend NBP by removing the corresponding ESM ensemble mean. The seasonal SOI index of DJF and MAM from NOAA is calculated by taking the mean of DJF and the mean of MAM.

### Definition of doubling sensitivity

**Fig. 8.**
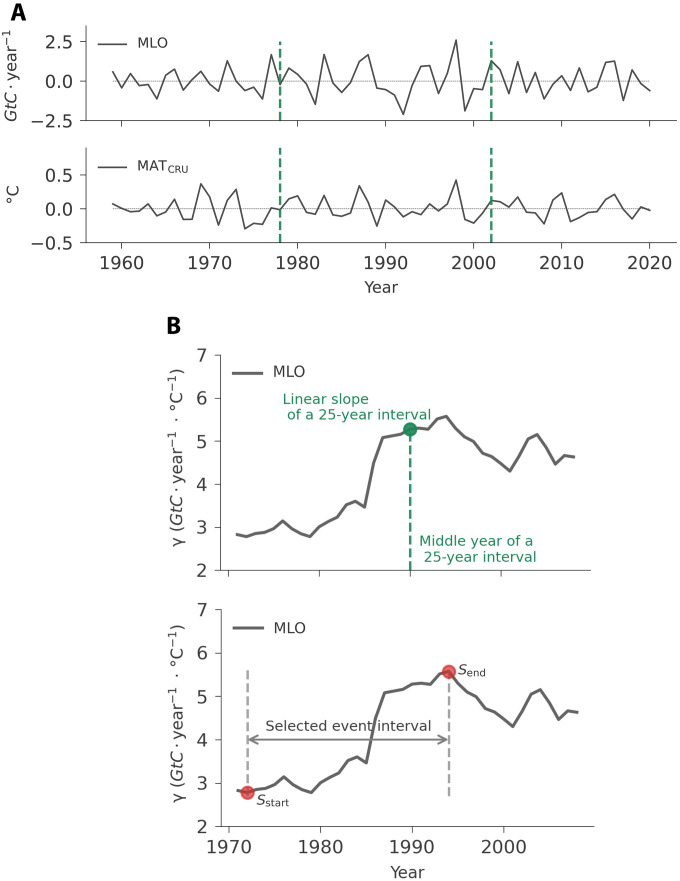
Schematic process to define the doubling sensitivity event. (**A**). Time series of AGR from MLO and tropical MAT from CRU. (**B**). We select a 25-year interval from the time-series of AGR and tropical MAT and calculate the simple linear regression slope *dAGR*/*dMAT*, and we use γ to represent the linear slope. Then, we plot all the calculated linear slopes in a 25-year moving window (top). Then, we select the event in the period 1972 to 1994 that shows a doubling sensitivity (bottom, two vertical dashed lines).

This study focuses on the doubling sensitivity of AGR to tropical MAT. We repeat the results from (*8*) by using the updated observation records from 1959 to 2020 (Fig. 8). Slightly differently from previous study (*8*), we remove the long-term trend for the whole time series of AGR and tropical MAT separately before selecting the 25-year interval and calculating the linear slope.

We define the doubling sensitivity event according to the sensitivity by using Mauna Loa and calculate its relative trend over a 25-year moving window. The selected event period is 23 years (Fig. 8B, bottom plot, 1972 to 1994, two vertical dashed lines). According to Eq. 2, the relative trend of the doubling sensitivity by using Mauna Loa is 100.5%.

$$d_{S}=100\%\left(S_{\text{end}}-S_{\text{start}}\right)/S_{\text{start}}$$ (2)

In the 25-year moving window, *S*_start_ and *S*_end_ are the sensitivity at the start and end of the event, respectively (Fig. 8B, bottom plot). *d_S_* is the relative slope of the doubling sensitivity event.

We then select similar events from five ESM large ensembles. First, for individual realizations, we calculate the sensitivity between NBP and tropical MAT (both variables have the respective ensemble means including their temporal trends removed) in a 25-year moving window. Note that there are a few calculated sensitivities (slopes) that have significance *P* > 0.05. We then select events from individual realizations that show a similar relative trend as in the sensitivity by using Mauna Loa, with all selected events having slopes with significance *P* < 0.05. We leave some allowance when selecting events in large ensembles to make sure to include the relevant events: The relative slope is in the range of 100.5 ± 2%, and the event period is in the range of 23 ± 2 years. Note that the event is not fixed in the period of 1972 to 1994 as in observations; rather, all periods are considered when selecting similar events from individual realizations.

### Interpretation of ensemble mean and spread

The ensemble mean and spread differ considerably between the ESMs, reflecting their different responses to external forcings and internal climate variability, e.g., (*23*, *29*, *83*–*85*). The five ESMs are all driven by the same set of CMIP6 historical forcings (*62*, *86*). The differences in the ensemble mean and spread arise from different physical processes, parametrizations, and numerical formulations among ESMs (*22*, *23*). For instance, the spread of NBP ensemble averages across models might be due to the different ecosystem responses and land use change in ESMs (*87*, *88*). Note that (i) the ensemble mean of all realizations in each ESM is considered mostly influenced by external forcing (*22*) and (ii) the ensemble spread within a given model is mainly influenced by internal climate variability through climate variables, time period, season, and location (*22*, *29*). The relatively stable ensemble spread, despite the increasing trend in the ensemble mean, suggests that external forcing has a small influence on internal climate variability (*29*).

### Statistical analysis

#### Sensitivity decomposition

In a simple linear regression, the slope is represented in (*89*)

$$\frac{\mathrm{dy}}{\mathrm{dx}}=\frac{\sum_{i=1}^{n}\left(y_{i}-\bar{y}\right)\;\left(x_{i}-\bar{x}\right)}{\sum_{i=1}^{n}{\left(x_{i}-\bar{x}\right)}^{2}}$$ (3)

*X* and *Y* are two variables, each including *n* samples and are represented as *x_i_* and *y_i_*. $\bar{x}$ and $\bar{y}$ are the means of the two variables, respectively. On the right side of Eq. 3, both divided by the standard deviation of *X* and *Y*, we have

$$\frac{\mathrm{dy}}{\mathrm{dx}}=\mathrm{cor}\left(X,Y\right)\;\frac{\mathrm{std}\left(Y\right)}{\mathrm{std}\left(X\right)}$$ (4)

*cor*(*X*, *Y*) represents Pearson’s correlation, and *std*(*X*) and *std*(*Y*) represent the standard deviation of *X* and *Y*, respectively. According to Eq. 4, the simple linear slope *dy*/*dx* is determined by the correlation *cor*(*X*, *Y*) multiplied by the relative variance *std*(*Y*)/*std*(*X*). In observations, we calculate the correlations between AGR and tropical MAT and the relative variance *std*(*AGR*)/*std*(*MAT*). In ESMs, we use NBP instead of AGR. Note that NBP has been multiplied by −1 when calculating the sensitivity, for sign consistency with AGR. We then calculate these metrics in a 25-year moving window and select the relevant doubling sensitivity event periods that are defined in the section Definition of doubling sensitivity. We approximate the contribution of the changes of each metric to the doubling sensitivity by their corresponding relative change. In the 25-year moving window, according to Eq. 5, the relative change equals the values at the end (*V*_end_) minus start (*V*_start_) of the event, and divided by the start (*V*_start_) value

$$\text{Relative change}=100\%\left(V_{\mathrm{end}}-V_{\text{start}}\right)/V_{\text{start}}$$ (5)

#### Variance decomposition

We decompose the global CO_2_ time series interannual variations into different spatial domains and calculate the contribution of each domain to the doubling sensitivity. According to (*89*), we have

$$\begin{array}{c} Var(X_{1}+X_{2}+X_{3}+\ldots X_{n})=Var(X_{1})+Var(X_{2})+Var(X_{3})+\ldots +Var(X_{n})\\ +2Cov(X_{1},X_{2})+2Cov(X_{1},X_{3})+\ldots +2Cov(X_{n-1},X_{n}) \end{array}$$ (6)

*X_i_* is a random variable. *Var*(*X_i_*) is the variance of *X_i_*. *Cov*(*X_i_*, *X_j_*) is the covariance between *X_i_* and *X_j_*. Covariance captures the linear dependence between two variables, and it measures the degree to which *X_i_* and *X_j_* vary together (*89*). Note that i and j denote two random numbers from 1 to n. 

We first decompose the global carbon sink to land and ocean

$$\mathrm{Var}\left(\text{Global}\right)=\mathrm{Var}\left(\text{Land}\right)+\mathrm{Var}\left(\text{Ocean}\right)+2\mathrm{Cov}\left(\text{Land},\text{Ocean}\right)$$ (7)

We then decompose the global land sink to various spatial domains: NH (N23.5° to N90°), Tro (N23.5° to S23.5°), and SH (S23.5° to S90°).

$$\begin{array}{c} \mathrm{Var}\left(\text{Land}\right)=\mathrm{Var}\left(\mathrm{NH}\right)+\mathrm{Var}\left(\mathrm{Tro}\right)\\ +\mathrm{Var}\left(\mathrm{SH}\right)+2\mathrm{Cov}\left(\mathrm{NH},\mathrm{Tro}\right)+2\mathrm{Cov}\left(\mathrm{Tro},\mathrm{SH}\right)+2\mathrm{Cov}\left(\mathrm{NH},\mathrm{SH}\right) \end{array}$$ (8)

We first calculate the decomposed variance in a 25-year moving window and then select the event period and calculate the contribution of each decomposed component to the total variance change during the doubling event: (i) For each component, we calculate the variance change (variance at the end of the event − variance at the start of the event), and (ii) we calculate the ratio of variance or covariance change of each decomposed component to the total variance change.

Note that the calculated variance and covariance cause minor uncertainties. In Eq. 8, the variance on the left side is slightly different from the sum of the decomposed variances and covariances on the right side. When using the left side as the total variance, then contribution of each decomposed variance/covariance on the right side adds up to around 93 to 99% in both observations and ESMs. Here, to avoid such minor uncertainties, we use the sum of all the decomposed variances and covariances as the total variance.
