# Supplementary material for: Enhanced global carbon cycle sensitivity to tropical temperature linked to internal climate variability
Source: Sci Adv. 2024 Sep 25;10(39):eadl6155. doi: 10.1126/sciadv.adl6155 (PMC11423872; doi:10.1126/sciadv.adl6155)
Supplement: Supplementary file 1 — Supplementary Text Figs. S1 to S10 Tables S1 to S8 [file sciadv.adl6155_sm.pdf]

Science Advances
AAAS

Supplementary Materials for

Enhanced global carbon cycle sensitivity to tropical temperature linked to internal climate variability

Na Li *et al.*

Corresponding author: Na Li, nali@bgc-jena.mpg.de

Sci. Adv. **10**, eadl6155 (2024)
DOI: 10.1126/sciadv.adl6155

This PDF file includes:

Supplementary Text
Figs. S1 to S10
Tables S1 to S8

Atmospheric inversions

The difference between the sensitivity based on Mauna loa and global carbon budget (as is shown in Fig. 1) suggests that the Mauna Loa AGR may contain substantial variations not related to fluxes but merely to atmospheric transport, that it may miss important parts of the flux signals due to its limited footprint, or that the calculation of a flux by year-to-year differentiation of the mole fraction (thereby assuming instantaneous atmospheric mixing) involves substantial artifacts.

The inversion considered so far ($CS57$, teal line as is shown in Fig. 1) only uses data from 3 stations which cover the entire 1959-2020 period. In order to investigate to which extent it misses part of the CO_2 flux variability, we compare it with an inversion based on further stations ($CS76$, light green) only available from 1976. The additional information in $CS76$ leads to smaller γ^T values, especially after about 1996. This uncertainty is to be expected due to missing inter-annual signals in $CS57$.

We also evaluated if the inversion is able to reproduce the growth rate implied by Mauna Loa observations (dotted light green compared to dotted black). We found inversion $CS57$ can well reproduce γ_{MLO}^T by transporting the corresponding surface fluxes forward with its atmospheric model (supplementary material Fig. S2).

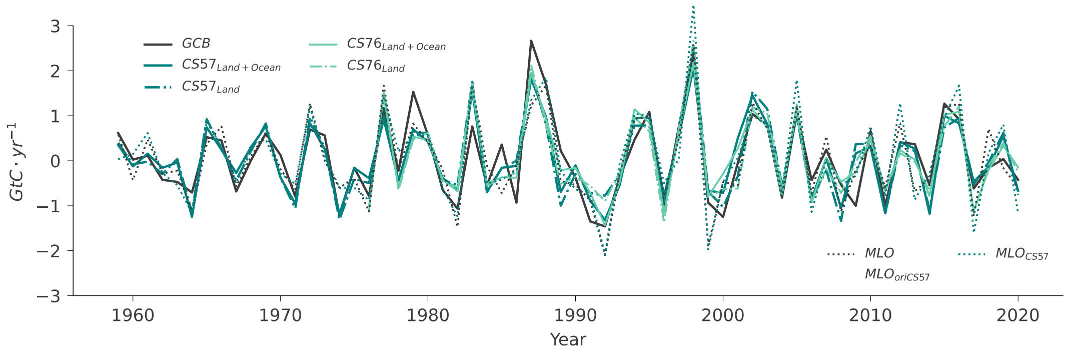

Figure S1: Time-series of different global CO_2 flux datasets: AGR (converted to a CO_2 flux assuming instantaneous atmospheric mixing) at Mauna Loa (MLO, black dotted line), global AGR based on multiple sites from the Global Carbon Budget 2021 (GCB, black filled line), and global CO_2 fluxes estimated by the Jena CarboScope atmospheric inversion using the stations Barrow, Mauna Loa, and South Pole covering almost the complete 1959-2020 period (s57Noc_STD1TneeI_v2022, here labelled $CS57_{Land+Ocean}$, solid teal), and an inversion based on further stations available since at least 1976 (s76oc_v2022, here labelled $CS76_{Land+Ocean}$, solid light green). We also included datasets only from the land flux estimated by the two inversions ($CS57_{Land}$ and $CS76_{Land}$, dash-dot lines of respective color).

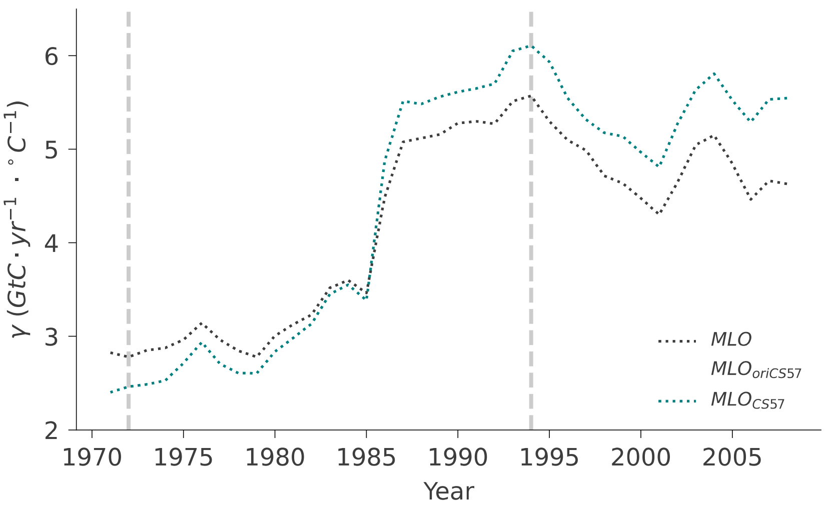

Figure S2: Inversion $CS57$ reproduced γ_{MLO}^{T} by transporting the corresponding surface fluxes forward with its atmospheric model (dotted teal line). Compared with the original MLO used (red dotted line), the prediction fits well. Note that the original MLO time-series used in prediction is pre-treated slightly different with NOAA (red dotted line compared with black dotted line).

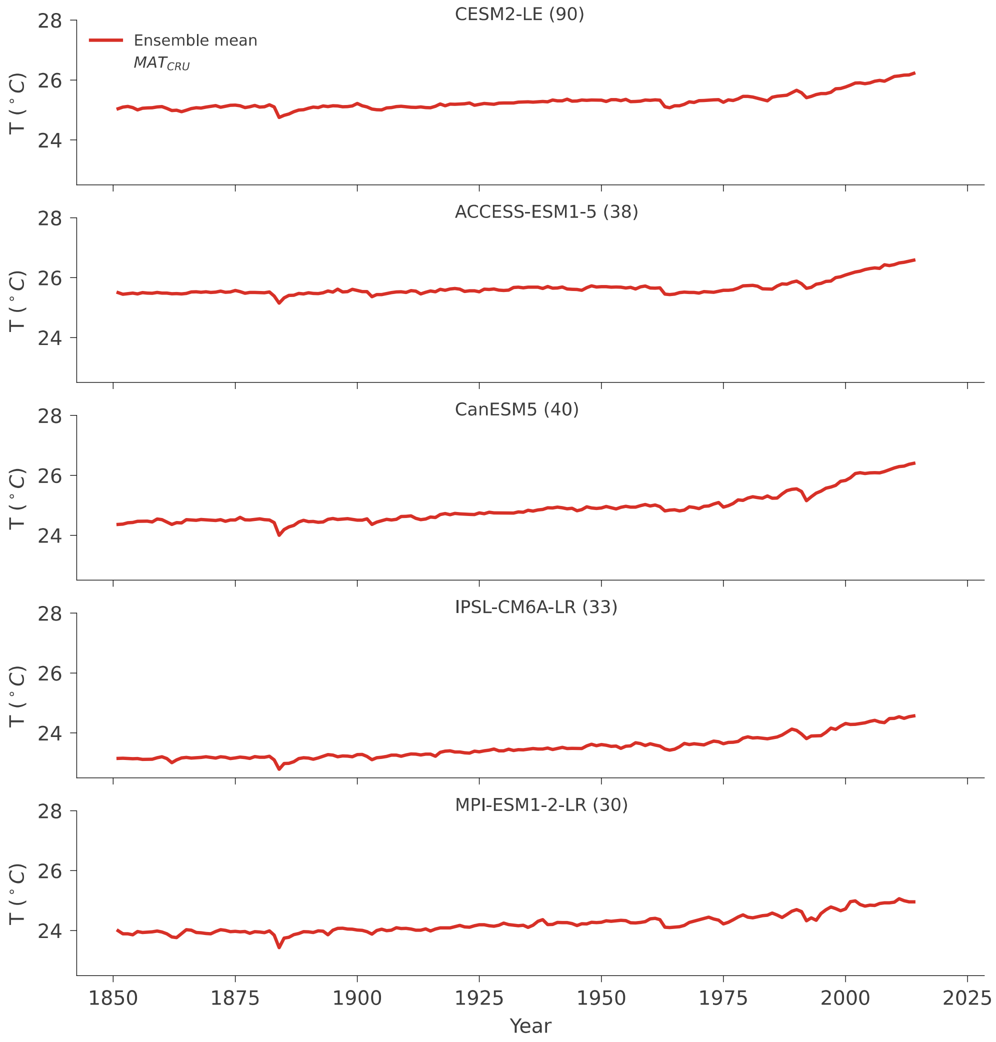

Figure S3: Tropical MAT time-series of five ESM large ensembles.

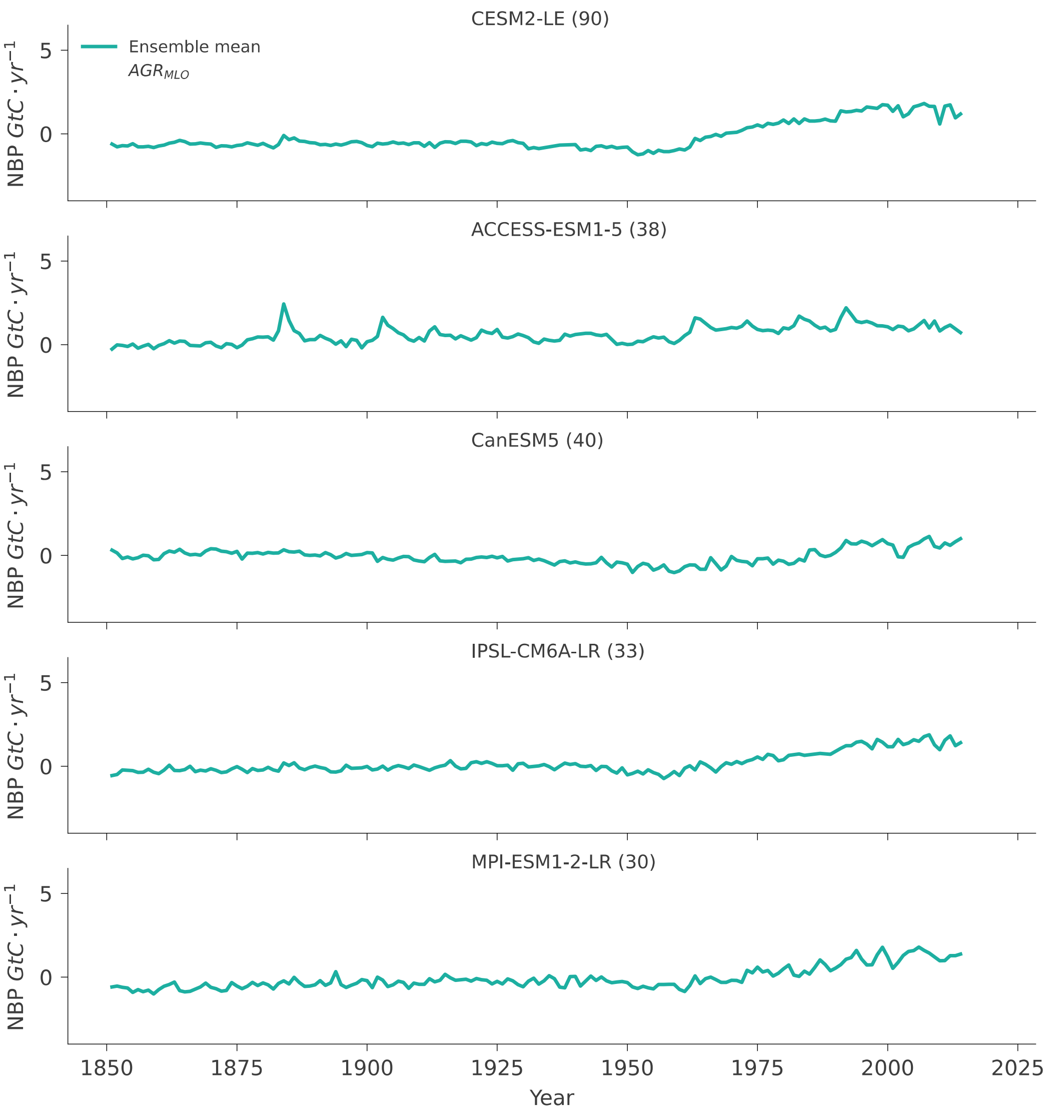

Figure S4: NBP time series from five ESM large ensembles. Individual realizations are plotted in light blue lines.

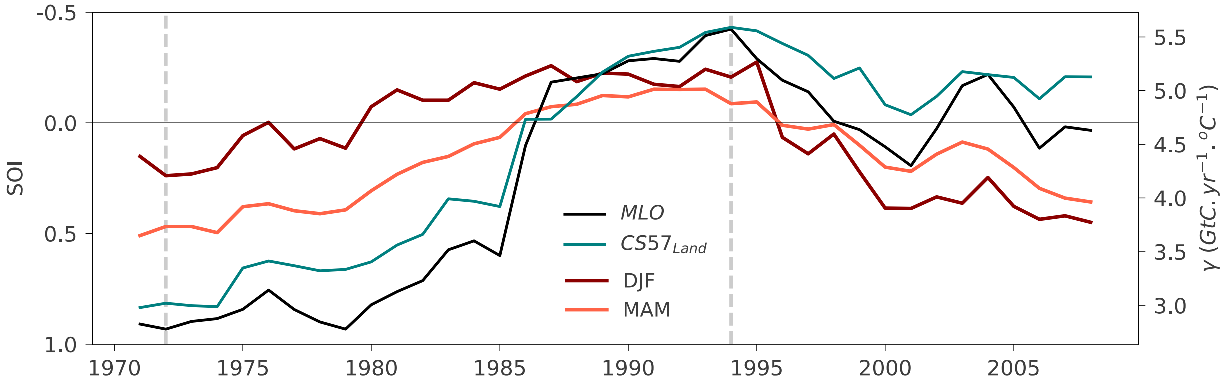

Figure S5: Seasonal SOI (DJF and MAM) in a mean 25-year moving window, and comparison with the sensitivity change based on Mauna Loa (MLO) and atmospheric inversion $CS57_{Land}$. Note that the left Y label is inverted.

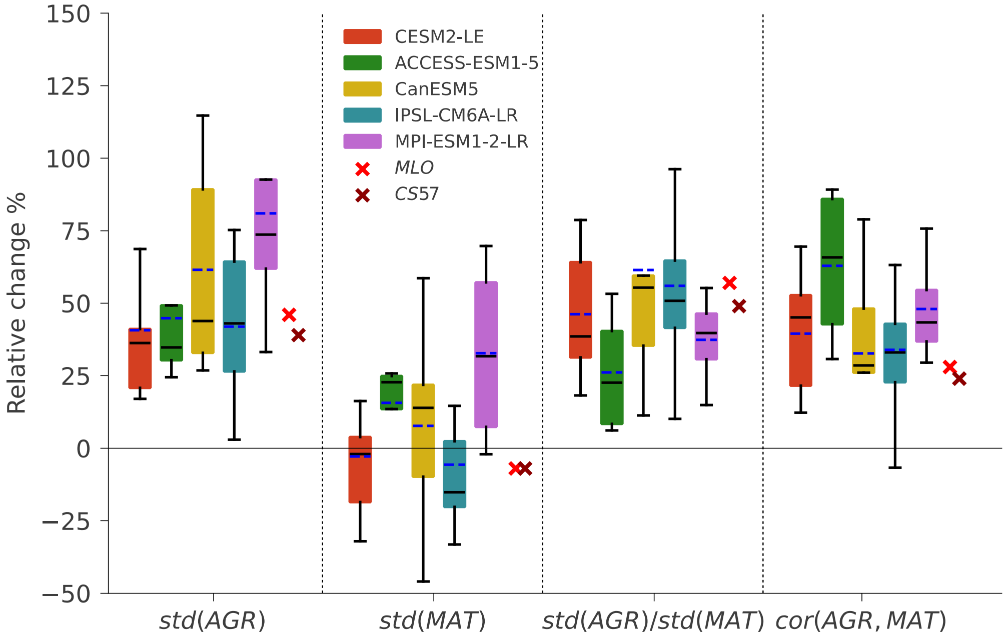

Figure S6: Relative change in each of the elements contributing to γ^T shown in Fig. 4 in the observation-based events (MLO and $CS57$) and across individual events in each of the ESMs considered. For each boxplot, the median is represented by black line, and the mean is the dashed blue line.

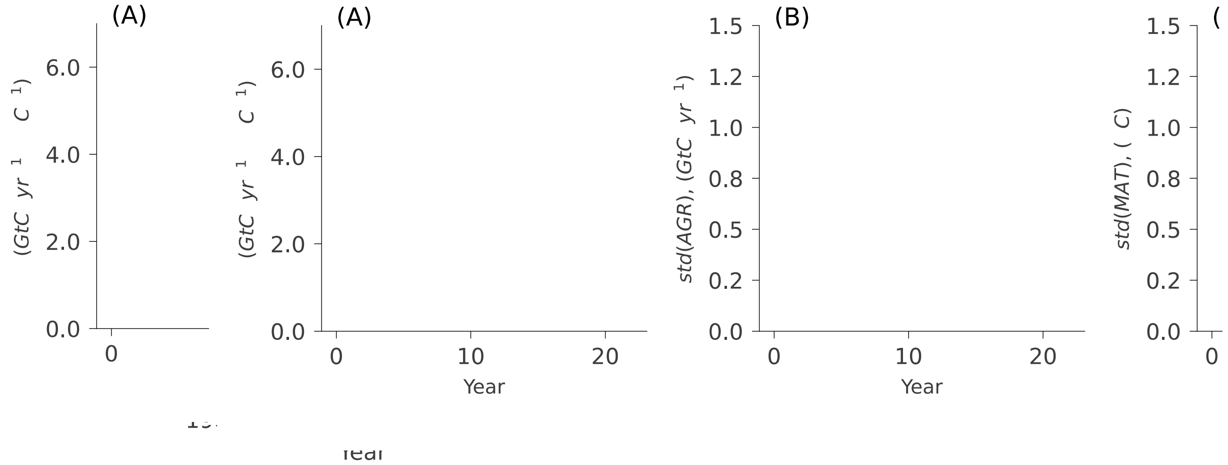

Figure S7: Variance decomposition of global CO_2 flux (land and ocean to atmosphere) from Jena CarboScope ($CS57_{Land}$), over 25-year moving windows. (A). Global CO_2 variance is decomposed to land and ocean. The variance of global AGR based on CO_2 mixing ratio Mauna Loa is shown in black. (B). Based on the variance change of each component in the doubling sensitivity period (variance at the end of the event minus variance at the start of the event, see Materials and methods), we plot the fraction contributed by each component in panel A to the global CO_2 variance change. The doubling sensitivity period is 1972-1994 (two vertical dashed lines). Note that variance is represented as "Var", and covariance as "Cov".

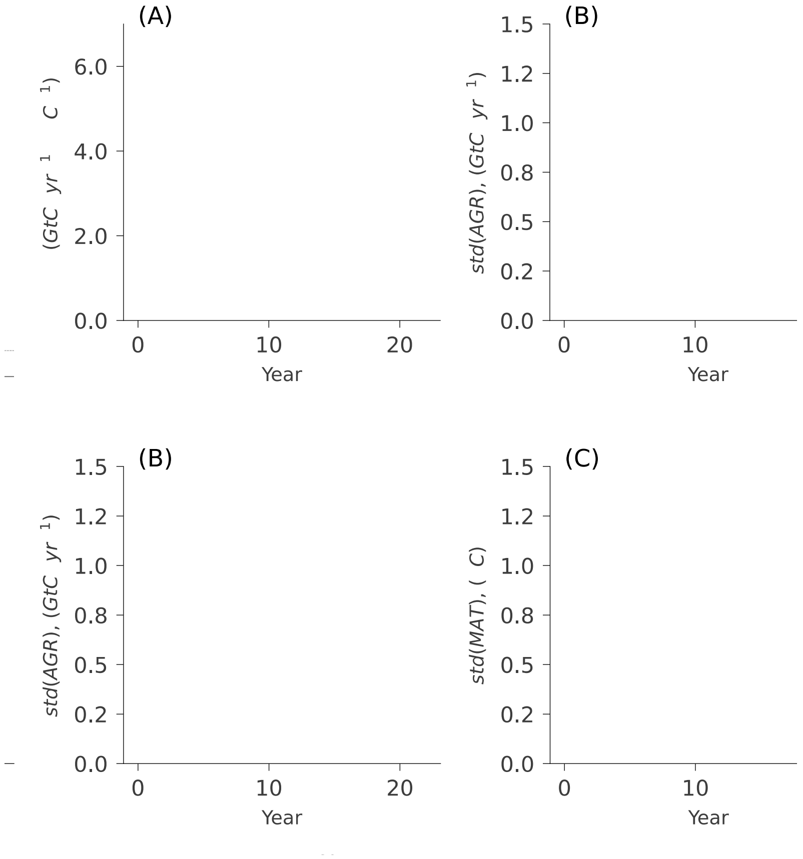

Figure S8: Variance decomposition of atmospheric inversions Jena CarboScope version $CS76$, covering period 1976-2020.

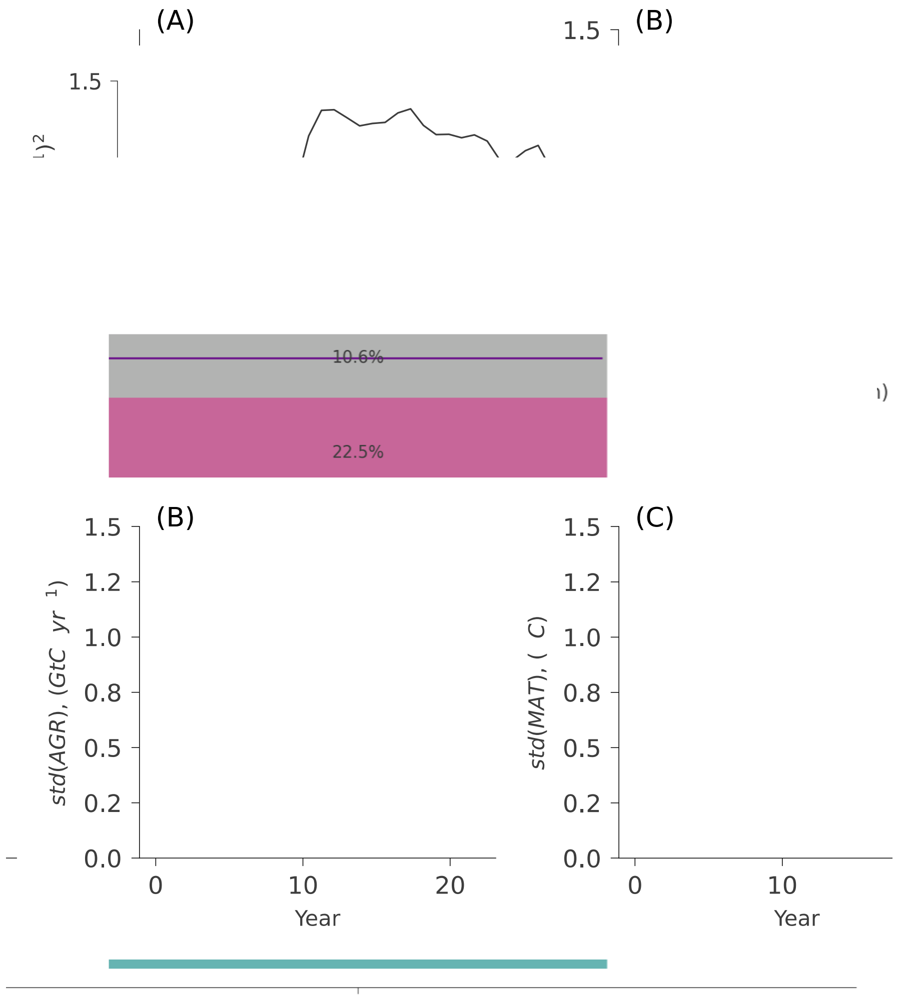

Figure S9: Variance decomposition of atmospheric inversions Jena CarboScope version $CS85$, covering period 1985-2020.

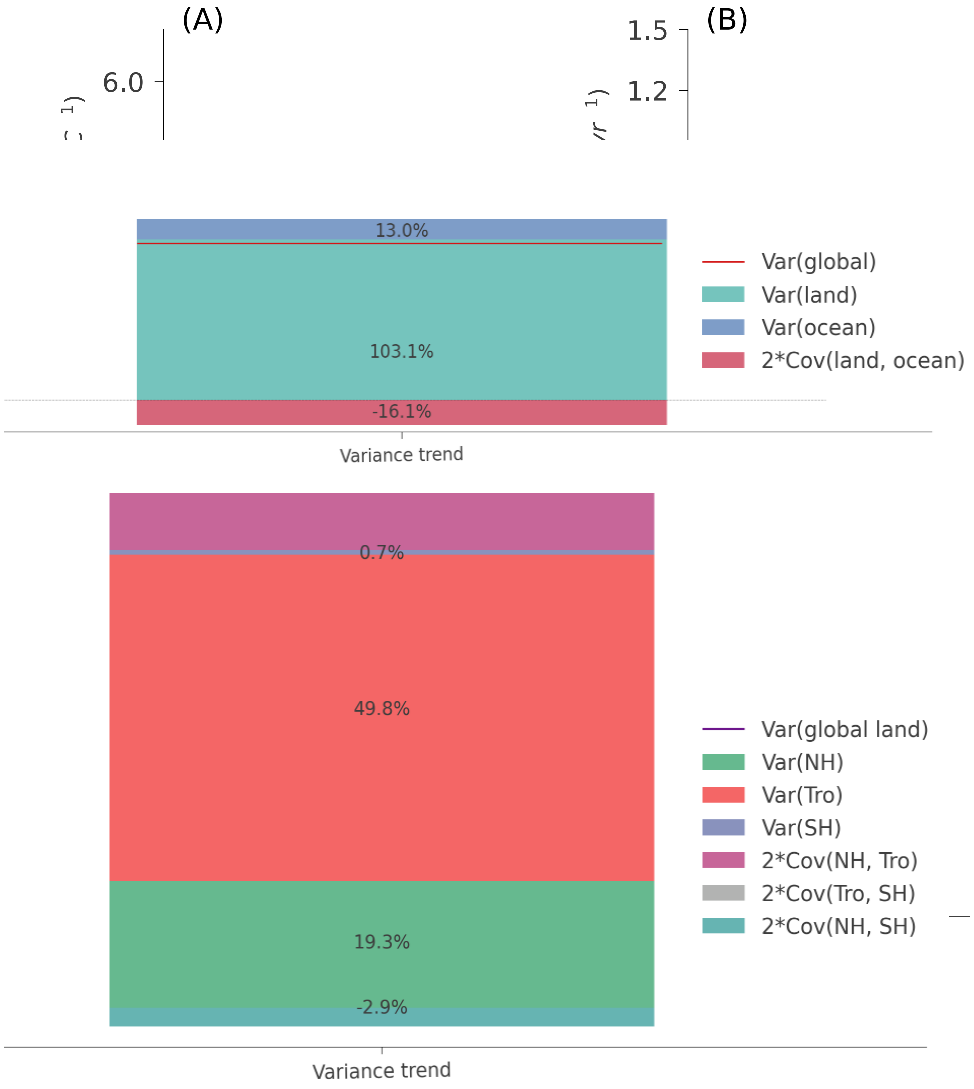

Figure S10: Variance decomposition of atmospheric inversions Jena CarboScope version $CS93$, covering period 1993-2020.

$\gamma\ (GtC \cdot yr^{-1} \cdot ^{\circ} C^{-1})$	1972	1994	2008
GCB	3.0	5.4	4.4
$CS57_{Land+Ocean}$	2.6	4.9	4.3
$CS57_{Land}$	3.0	5.6	5.1
$CS76_{Land+Ocean}$	-	4.8	4.0
$CS76_{Land}$	-	5.5	4.8
MLO	2.8	5.6	4.6

Table S1: Changes in the sensitivity of global CO_2 time-series variations to tropical MAT based on linear regression slope over 25-year moving windows. We use γ^T to represent the sensitivity. The sensitivity is calculated for different datasets: AGR at Mauna Loa (MLO) and global AGR based on multiple sites from the Global Carbon Budget 2021 (GCB). We compare these with different estimates of carbon cycle sensitivity to tropical MAT by the Jena CarboScope atmospheric inversion (two versions, $CS57$ and $CS76$): the sensitivity of the global aggregated sink in land and ocean ($CS57_{Land+Ocean}$, $CS76_{Land+Ocean}$), the land sink only ($CS57_{Land}$, $CS76_{Land}$), and finally, for $CS57$, we evaluate whether $CS57$ can reproduce γ^T_{MLO} by transporting the corresponding surface fluxes forward with an atmospheric model. 1972, 1994 are the start and end of the doubling sensitivity period, and 2008 is the last year over the 25-year moving window.

γ^T	SOI DJF	SOI MAM
γ^T_{MLO}	-0.15	-0.83
γ^T_{GCB}	-0.42	-0.92
$\gamma^T_{CS57Land+Ocean}$	-0.17	-0.85
$\gamma^T_{CS57Land}$	-0.06	-0.79

Table S2: Correlation of different time series of γ^T with 25-year smoothed SOI.

Relative change (%)	AGR_{MLO}	AGR_{CS57}
std_{AGR}	46	39
std_{MAT}	-7	-7
std_{AGR}/std_{MAT}	57	49
$cor(AGR, MAT)$	28	24

Table S3: Relative change of standard deviation and correlation of AGR, during the doubling sensitivity event. AGR is from Mauna Loa (MLO) and atmospheric inversion $CS57$. Note that the relative change is calculated according to Equation 5 in Section Materials and methods.

Relative change (%)	1	2	3	4	5	6	7	8	9	10	11	12	13	14	15	16
Std_{NBP}	34	38	69	44	21	36	17	21	142	21	40	40	36	43	18	32
Std_{MAT}	-2	-19	12	16	-11	-2	-1	-7	49	-32	-19	1	-24	11	-19	0
Std_{AGR}/Std_{MAT}	36	70	51	24	36	39	18	30	62	78	73	38	79	29	45	32
$Cor(AGR, MAT)$	48	18	33	62	47	44	69	54	23	13	16	46	12	56	39	52

Table S4: Relative change of standard deviation and correlation of NBP in 16 events of CESM2-LE, during the doubling sensitivity event. Note that the relative change is calculated according to Equation 5 in Section Materials and methods.

Relative change (%)	1	2	3	4
Std_{AGR}	32	24	85	37
Std_{MAT}	25	-9	21	26
Std_{AGR}/Std_{MAT}	6	36	53	9
$Cor(AGR, MAT)$	89	47	31	85

Table S5: Relative change of standard deviation and correlation of NBP in 4 events of ACCESS-ESM1-5, during the doubling sensitivity event. Note that the relative change is calculated according to Equation 5 in Section Materials and methods.

Relative change (%)	1	2	3	4	5
Std_{AGR}	27	89	115	33	44
Std_{MAT}	14	22	59	-46	-10
Std_{AGR}/Std_{MAT}	11	55	35	146	59
$Cor(AGR, MAT)$	79	29	48	-18	26

Table S6: Relative change of standard deviation and correlation of NBP in 5 events of CanESM5, during the doubling sensitivity event. Note that the relative change is calculated according to Equation 5 in Section Materials and methods.

Relative change (%)	1	2	3	4	5	6	7	8
Std_{AGR}	45	75	64	41	64	11	32	3
Std_{MAT}	-2	-19	49	15	-16	-25	-14	-33
Std_{AGR}/Std_{MAT}	48	115	10	23	96	48	53	54
$Cor(AGR, MAT)$	36	-7	81	63	3	35	31	29

Table S7: Relative change of standard deviation and correlation of NBP in 8 events of IPSL-CM6A-LR during the doubling sensitivity event. Note that the relative change is calculated according to Equation 5 in Section Materials and methods.

Relative change (%)	1	2	3	4
Std_{AGR}	76	72	143	33
Std_{MAT}	53	11	70	-2
Std_{AGR}/Std_{MAT}	15	55	43	36
$Cor(AGR, MAT)$	76	29	39	47

Table S8: Relative change of standard deviation and correlation of NBP in 4 events of MPI-ESM1-2-LR, during the doubling sensitivity event. Note that the relative change is calculated according to Equation 5 in Section Materials and methods.
